# Outcomes of a residential respite service for homeless people with tuberculosis in London, UK: a cross-sectional study

**DOI:** 10.1177/17579139221093544

**Published:** 2022-05-04

**Authors:** L Crosby, D Lewer, Y Appleby, C Anderson, A Hayward, A Story

**Affiliations:** Collaborative Centre for Inclusion Health, UCL, London, UK; Research Department of Primary Care and Population Health, UCL, London, UK; Collaborative Centre for Inclusion Health, University College London, London, UK; National Infections Service, Public Health England, London, UK; Find & Treat, University College London Hospitals, London, UK; National Infections Service, Public Health England, London, UK; Collaborative Centre for Inclusion Health, UCL, London, UK; Collaborative Centre for Inclusion Health, UCL, London, UK; Find & Treat, University College London Hospitals, London, UK

**Keywords:** tuberculosis, homeless, residential respite, elimination, inclusion health, social exclusion, treatment completion, vulnerable populations

## Abstract

**Background:**

Many countries are seeking to eliminate tuberculosis (TB), but incidence remains high in socially excluded groups such as people experiencing homelessness. There is limited research into the effectiveness of residential respite services (RRS), which provide accomodation and social and clinical support for homeless people with active TB.

**Methods:**

We used a register of all cases of TB diagnosed in London between 1 January 2010 and 3 October 2019 to compare characteristics and outcomes of patients treated in an RRS with patients receiving standard care. The primary outcome was successful treatment completion. We used logistic regression to compare likelihood of completing treatment, and simulation to estimate the absolute change in treatment completion resulting from this service.

**Results:**

A total of 78 homeless patients finished an episode of TB treatment at the RRS. Patients treated in the RRS were more likely than patients treated in standard care to have clinical and social risk factors including drug resistance, history of homelessness, drug or alcohol use, and need for directly observed therapy. After adjusting for these factors, patients treated in the RRS had 2.97 times the odds of completing treatment (95% CI = 1.44–6.96). Treatment ended in failure for 8/78 patients treated in the RRS (10%, 95% CI = 5%–20%). We estimated that in the absence of the RRS, treatment would have ended in failure for 17/78 patients (95% CI = 11–25).

**Conclusion:**

The residential respite service for homeless TB patients with complex social needs was associated with better treatment outcomes.

## What is the Key Question?

How does a residential respite service (RRS) affect the likelihood of TB treatment completion for homeless TB patients in London?

## What is the Bottom Line?

Patients treated in the RRS had higher prevalence of clinical and social risk factors for TB treatment failure than patients treated in standard care. The crude risk of TB treatment failure was similar in the two settings. After adjusting for clinical and social risk factors, patients treated in the RRS were almost three times more likely to complete TB treatment.

## Why Read On?

Improving TB outcomes among socially excluded groups including people experiencing homelessness is challenging and is central to elimination of TB in low incidence countries. These results show that an RRS is associated with improved TB treatment outcomes among these groups.

## Introduction

Tuberculosis (TB) remains a major global health problem despite substantial reductions in incidence,^1^ with an estimated 10 million new cases in 2018.^2^ While antibiotic treatment is effective, the toxicity and duration are obstacles to treatment completion. Non-adherence is common and in 2017 an estimated 15% of patients who began TB treatment did not complete.^2^ This leads to further transmission, preventable deaths, and the development of antibiotic resistance.

TB is associated with poor and overcrowded living conditions.^3,4^ In low incidence countries, TB is increasingly concentrated among groups with social risk factors including those with experience of homelessness, prison, alcohol dependence, and illicit drug use.^5,6^ Global,^7^ regional,^8,9^ and national strategies^10^ highlight these socially excluded groups as priorities for TB elimination. Homelessness is one of the most important risk factors for TB infection and transmission.^11^ Homeless people may have increased exposure, delayed diagnosis, prolonged infectivity due to late diagnosis, lower adherence to treatment, and higher risk of complex and drug resistant disease.^12^ Together, these factors can lead to increased risk of treatment failure and multiple episodes of TB.^13–17^ In addition, some homeless people in the UK have ‘no recourse to public funds’ due to their migration status, meaning they cannot access welfare benefits or assistance with housing.

Clinical guidance in the UK recommends that people with active TB – including those with no recourse to public funds – should be provided with state-funded accommodation.^18^ However, the lack of an agreed national pathway means such patients are at risk of being discharged to the street following hospitalisation for TB treatment.^19^ Where accommodation is provided it is often in ‘bed and breakfast’ style lodging, lacking social and clinical support. Some services were previously established to provide integrated accommodation and social support to homeless people but have long since been closed.^20^ Today, models for supporting TB patients with complex social needs include ‘ad hoc’ social support provided separately from accommodation, or service-level agreements between discharging hospitals and local housing teams to provide rapid access to accommodation, without in-house social support.^5^

Most research into approaches to improving outcomes for homeless TB patients focuses on patient behaviour, including educational interventions, psychological support, incentives, and directly/video observed therapy (DOT/VOT).^15,16,21^ Limited research has investigated the effectiveness of interventions that aim to improve housing and other material and social factors. We are aware of two previous studies of the outcomes of housing interventions for homeless TB patients in South Korea^22^ and the US,^23^ which both suggested improved treatment outcomes but were limited by their ability to account for differences in patients’ clinical characteristics. One observational study found that homeless people in South Korea who received an enhanced housing package (including food and social support) had improved treatment completion, relative to treatment as usual.^22^ Another study found that homeless people in California placed in residential treatment programmes had improved treatment completion rates compared with historical and neighbouring locations.^23^

### Olallo House: a residential respite service in London, UK

TB patients in London are usually managed in the community at outpatient clinics. Patients are assessed regularly and clinicians record risk factors including homelessness, drug and alcohol use, comorbidities such as HIV, and mental health problems. Directly Observed Treatment or Video Observed Treatment is sometimes provided for patients who have a high risk of treatment failure.^21^ However, treatment failure is common in groups with these risk factors, particularly those with no recourse to public funds.

In response to these problems, a partnership of NHS and charitable organisations set up a residential respite service (RRS) in central London, UK, in 2010. The RRS is located in Olallo House, a ‘safe house’ for vulnerable migrants run by the charity Saint John of God Hospitaller Services. It aims to support homeless TB patients with no recourse to public funds; facilitate safe and timely discharge from hospital; support TB treatment; provide accommodation; and provide comprehensive support including psychological help and support for drug and alcohol dependency. The staff team provides support for a range of social needs, seeking to enable long-term recovery, access to housing and employment, and independent living. To our knowledge this is the only contemporary UK example of a dedicated residential intervention providing comprehensive health and social support to TB patients with no recourse to public funds and complex social needs.

We aimed to compare the characteristics and treatment outcomes of patients treated at the RRS with patients treated in standard care, and to estimate the association between treatment at the RRS and treatment outcomes.

## Method

We did a cross-sectional analysis using linked routine surveillance data from Public Health England (now known as the UK Health Security Agency) and from the RRS. We compared cases treated in the RRS with all other cases of TB notified in London. Descriptive analysis compared the demographic, clinical and social characteristics of the two groups. We used logistic regression to estimate the association between support at the RRS and treatment outcomes.

### Dataset and sample

We used data from the London TB Register (LTBR), a routine surveillance database maintained by Public Health England, which includes information on all cases of TB notified by medical practitioners in London. In the UK, TB is a statutorily notifiable disease and LTBR collects data on patient demographics, disease factors such as site, drug sensitivities and previous TB treatment history, and social risk factors for all cases diagnosed or managed by TB clinics in London.^24,25^ Data are entered to LTBR by clinic staff. Data on drug resistance is added directly from reference laboratory reports. We extracted demographic, clinical, and social information from LTBR for all cases aged 18 or older notified between 1 January 2010 and 3 October 2019 (26,297 cases). Each record represented a unique ‘treatment notification period’, which begins upon notification and ends when a final outcome (treatment completion, death, loss to follow-up, or transfer to another clinic) is recorded.

We extracted data from the RRS including a unique individual identifier that was common with LTBR, the dates of residence at the RRS, and details of social risk factors such as homelessness. We linked this data to the LTBR data using the unique identifier and flagged cases that were resident at the RRS during their notification period (89 cases). Five patients treated in the RRS had multiple episodes, but each had only one episode of treatment at the RRS (i.e. the other episodes were in standard community services), and in all such instances the episode in the RRS was the most recent episode.

**Figure 1:**
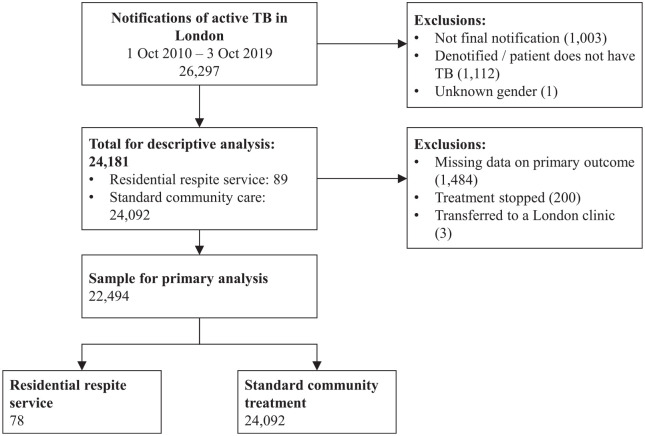
Flow-chart showing derivation of the study sample

For descriptive analysis, we excluded cases where the patient had a later episode of TB (1003 cases); cases that were ‘de-notified’ due to misdiagnosis of TB (1112 cases); and one case with unknown gender (see Figure 1). Further exclusions were made for logistic regression analyses, due to missing variables specific to each outcome (see below, and Figure 1).

### Outcomes

Our primary outcome variable was a binary flag indicating treatment completion. Those who died, were lost to follow-up, or were flagged as ‘transferred out’ to non-London clinics without further information were considered to have not completed treatment. Those with missing outcome data (1484 cases), whose TB treatment was stopped (200 cases) or who were transferred to a clinic outside of London (three cases) were excluded from primary regression analysis. Following these exclusions, 22,494 individuals (of which 78 treated in the RRS) were included in regression analysis.

Our secondary outcome was death during the notification period; cases where death was flagged in LTBR (TB as direct, contributing or incidental cause) were assigned this outcome. We exclude cases that were lost to follow-up, had no outcome information, or who were transferred out (2992 cases).

### Covariates

We selected potential confounders based on an a-priori logic model (see Supplementary information). Demographic covariates were (1) age, (2) sex, and (3) ethnic group (Asian, White, Black, other, or unknown). Clinical covariates were (1) the patient’s sputum smear status; (2) the site of disease, coded as two non-exclusive binary variables showing (a) whether the patient had pulmonary disease, and (b) whether the patient had disease at a ‘complex site’ including miliary, central nervous system, or disseminated TB; (3) drug resistance, from clinician input and verified by reference laboratory tests, coded into three levels based on the maximum resistance indicated in either source: (a) fully sensitive or unknown, (b) isoniazid mono-resistant, (c) rifampicin resistant or multidrug resistant (MDR). Social covariates were (1) history of homelessness, (2) history of drug use, (3) history of prison, (4) alcohol dependence (recorded as concerns about the patient’s ability to self-administer treatment affected by alcohol), and (5) whether a need for DOT was recorded. DOT may be recommended for different reasons but often relates to social barriers to care and we therefore used it as a marker of social exclusion.

### Statistical analysis

We compared patients treated in the RRS with patients treated in standard care in terms of demographic, clinical, and social variables. We then used logistic regression to estimate the association between the outcomes and the location of treatment (the RRS or standard community care), adjusting for demographic, clinical, and social covariates. We then conducted a simulation to estimate how many treatment completions would be experienced among patients treated in the RRS if they were treated in standard community services. In this simulation, we fit a logistic regression model on the whole sample with treatment completion (primary outcome) as the dependent variable and the same independent variables as above but excluding the location of treatment. We then used this model to generate 1000 simulations of the primary outcome (i.e. treatment success/failure) for the 78 RRS patients with data on treatment completion; interpretable as scenarios in which these patients were treated in standard community care. We reported the .025, .5 and .975 quantiles of the number of treatment completions.

### Missing data

Some patients did not have outcome data (e.g. due to ongoing treatment) and we excluded these patients from analysis. Where information on social risk factors (history of homelessness, drug use, prison, or alcohol dependence) was missing, we coded the variable as ‘missing’. We conducted a sensitivity analysis to assess the possible extent of bias resulting from this missing data. We created two scenarios: (1) imputing data for missing social risk factors for patients treated in the RRS as the presence of risk factors, and for patients treated in standard community care as the absence of risk factors and (2) the reverse scenario, imputing missing social risk factors for patients treated in the RRS as the absence of risk factors, and for patients treated in standard community care as the presence of risk factors. We reported the primary outcome in these scenarios. Data were complete for other variables, apart from sex, which was missing for one patient (who we excluded from analysis).

Analysis was performed in R version 3.5.2.

## Results

A total of 24,181 patients meeting the inclusion criteria were notified to LTBR between 1 January 2010 and 3 October 2019, of which 89 were treated at the RRS.

**Figure 2:**
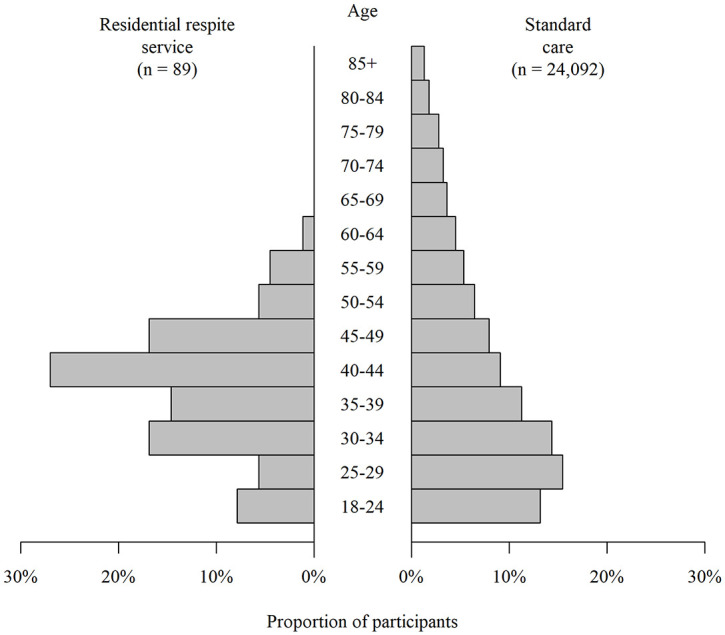
Age distribution of study participants

Patients treated in the RRS were more likely to be male, of White ethnicity, and born outside of the UK. Although the median ages were similar, the age distribution was different. Those treated in the RRS had a narrower distribution with an older modal age (Figure 2). Patients treated in the RRS were more likely to be sputum-smear positive, have pulmonary TB, to have been a hospital inpatient during their TB episode, to have isoniazid mono-resistant, rifampicin resistant or MDR, and more likely to require management via DOT. RRS residents were much more likely to have experienced homelessness, imprisonment, drug use, or to currently use alcohol to an extent that it affects their ability to self-administer treatment (Table 1).

**Table 1:** Demographic, clinical, and social characteristics of patients with diagnosed TB in London, 2010–2019

Variable		Residential respite service	Standard community care
Total		89 (100%)	24,092 (100%)
Demographic characteristics			
Age	Median (IQR)	40 (34–45)	37 (28–51)
Female sex		3 (3.37%)	9845 (40.86%)
Ethnicity	Asian	5 (5.62%)	10,977 (45.56%)
	Black	12 (13.48%)	6055 (25.13%)
	White	49 (55.06%)	3,107 (12.9%)
	Other	23 (25.84%)	3722 (15.45%)
	Unknown	0 (0%)	231 (0.96%)
Clinical characteristics			
Sputum smear positive		46 (51.69%)	2545 (10.56%)
Pulmonary TB		82 (92.13%)	11,007 (45.69%)
Miliary, CNS or disseminated TB		3 (3.37%)	1078 (4.47%)
Hospitalised		69 (77.53%)	7586 (31.49%)
Drug sensitivity	Fully sensitive	73 (82.02%)	22,756 (94.45%)
	Isoniazid mono-resistant	8 (8.99%)	927 (3.85%)
	Rifampicin resistant or MDR	8 (8.99%)	409 (1.7%)
Social characteristics			
History of drug use	No	62 (69.66%)	22,327 (92.67%)
	Yes	23 (25.84%)	886 (3.68%)
	Missing	4 (4.49%)	879 (3.65%)
History of homelessness	No	13 (14.61%)	22,521 (93.48%)
	Yes	76 (85.39%)	844 (3.5%)
	Missing	0 (0%)	727 (3.02%)
History of prison	No	71 (79.78%)	22,626 (93.91%)
	Yes	13 (14.61%)	664 (2.76%)
	Missing	5 (5.62%)	802 (3.33%)
Alcohol	No	47 (52.81%)	21,599 (89.65%)
	Yes	40 (44.94%)	901 (3.74%)
	Missing	2 (2.25%)	1592 (6.61%)
Need for DOT		79 (88.76%)	2838 (11.78%)
Outcomes			
Treatment completion	Yes	70 (78.65%)	20,046 (83.21%)
	No	8 (8.99%)	2370 (9.84%)
	Incomplete	11 (12.36%)	1676 (6.96%)
Death	Yes	2 (2.25%)	871 (3.62%)
	No	71 (79.78%)	20,245 (84.03%)
	Incomplete	16 (17.98%)	2976 (12.35%)

TB: tuberculosis; MDR: multidrug resistant; DOT: directly observed treatment; IQR: interquartile range; CNS: central nervous system.

### Primary outcome

A total of 22,494 patients had a valid primary outcome measure at the end of the notification period, of whom 78 were treated in the RRS. A similar proportion of patients treated in the RRS and in standard community care completed treatment (approximately 90% in each setting) and the crude odds ratio was 1.03 (95% CI = 0.53–2.34). After adjusting for demographic, social, and clinical variables, the odds ratio was 2.97 (95% CI = 1.44–6.96). The results are shown in Table 2. Detailed results of the regression model, including coefficients for covariates, are shown in Supplementary information. In sensitivity analysis of missing data, the fully adjusted odds ratio was 2.87 (95% CI = 1.40–7.03) in the first scenario and 3.00 (95% CI = 1.48–6.97) in the second scenario, suggesting limited potential bias from missing data in social risk factors. We also observed strong associations between missing data and treatment failure (see Supplementary information), which may suggest a process in which covariate data is less likely to be recorded for patients who do not complete treatment.

**Table 2:** Association between treatment in a residential respite services and outcomes (treatment completion and death)

	Odds ratio (95% confidence interval)
Primary outcome: treatment completion	
Unadjusted	1.03 (0.53–2.34)
Adjusted for demographic variables	1.29 (0.65–2.92)
Adjusted for demographic and clinical variables	1.67 (0.84–3.80)
Fully adjusted (demographic, clinical, and social variables)	2.97 (1.44–6.96)
Secondary outcome: death	
Unadjusted	0.65 (0.11–2.09)
Fully adjusted	0.37 (0.06–1.31)

### Secondary outcome

We were able to ascertain whether death occurred for 21,189 patients, of whom 73 were treated in the RRS. Of those treated in standard care, 871/21,116 (4.1%) died, while for those treated in the RRS 2/73 died (2.7%). The fully adjusted odds ratio for death during treatment comparing patients treated in the RRS with patients treated in standard community care was .37 (95% CI = 0.06–1.31).

### Simulation

Among patients treated in the RRS, 8/78 episodes ended in treatment failure. Based on simulation we estimate that in the absence of the RRS, 17/78 episodes (95% CI = 11–25) would have ended in failure. This suggests that the RRS was associated with a halving of the number of treatment failures.

## Discussion

Our results suggest that a residential respite service with housing and care is associated with reduced risk of TB treatment failure for patients with social risk factors and clinically complex disease (including multidrug resistance).

Risk factors for treatment failure were common in the RRS cohort. Despite this, patients treated at the RRS service had similar crude probability of completing treatment to patients in the community. The regression modelling and simulation suggest that risk of treatment failure for these patients would have more than doubled in standard community care, increasing risk of mortality, hospital re-admission, onward transmission, and development of multidrug resistance. We did not find evidence of a difference in mortality risk but our analysis lacked power.

There are several possible reasons why treatment at the RRS was associated with better outcomes. First, the RRS provided accommodation during TB treatment. People with social risk factors such as homelessness are often discharged from hospital into inadequate living conditions, including rough sleeping.^26^ Attending outpatient appointments and adhering to antibiotic regimens can be challenging in these conditions. Second, the RRS at Olallo House provides DOT for all residents and achieves high rates of treatment fidelity which may not be the case for patients treated under DOT in the community.^21^ Third, the RSS aims to improve social outcomes including helping residents to find work, live independently, and reconcile with families and home communities, and this may improve treatment success.

Our findings are the first that we know of to estimate the effect of a housing or residential service package on TB treatment completion among people legally unable to access state housing support. Our results are similar to those found by a study of patient in South Korea, which estimated that an intervention including housing, meals, DOT and case management was associated with an increased likelihood of treatment success (adjusted OR: 4.19, 95% CI =1.63–10.80).^22^ Our study also adds to an emerging body of literature that demonstrates the importance of intermediate or ‘step-down’ care upon discharge from hospital for improving health outcomes for people with social risk factors.^27,28^ We used a comparison group of patients treated in standard services, something not undertaken in previous studies of similar populations.^23^ A key strength of our study is the use of a routine dataset, which allowed access a large, well-characterised sample, including demographic, clinical and social information. Previous evaluations have not been able to adjust for clinical and social characteristics of participants.^22^

The study also has several limitations. We focused on TB treatment outcomes and did not capture other potential benefits of the RRS, including reduced delays to hospital discharge, reduced risk of re-admission, reduced onward transmission, and broader social benefits. We identified some issues with data quality, and particularly in under-recording of social risk factors. For example, routine data in LTBR show that 85% of patients treated in the RRS had experienced homelessness, but all RRS residents are homeless on entry. Social risk factors may also be under-recorded for patients treated in standard community care. There may also be residual confounding where variables recorded in LTBR do not fully reflect differences between the groups. For example, patients treated in the RRS have usually experienced long periods of sleeping rough immediately prior to their episode of TB, while patients identified as homeless in standard community care may have a range of experiences, including shorter periods and less severe forms of homelessness such as sofa-surfing.

Achieving successful treatment outcomes for homeless people with no recourse to public funds can be challenging and expensive. The mean length of stay at the RRS within our cohort was 230 days, which at a cost of £90 per day (the amount paid by commissioners of the service at the time of publication) equates to £20,700 per person. This is lower than the costs noted in other examples of individuals with TB and no resource to public funds, which have shown that costs of hospital inpatient care with DOT and additional case support can be over £170,000.^19^ Comparing the costs and outcomes of the dedicated RRS investigated here against other ad hoc forms of support – such as provision of social support outside of the residential setting, or service-level agreements between secondary care and local housing teams^5^ – is beyond the scope of this article but would be a fruitful avenue for further research.

## Conclusion

Incidence of TB remains high in socially excluded groups, even while incidence of the disease in the general population has fallen over the past decade.^6^ The findings reported here provide evidence that treatment in an RRS can improve treatment success for homeless people with no recourse to public funds. Patients treated in the RRS had higher prevalence of clinical and social risk factors for TB treatment failure than patients treated in standard care. The crude risk of TB treatment failure was similar in the two settings. After adjusting for clinical and social risk factors, patients treated in the RRS were more likely to complete TB treatment.

Improving TB outcomes among socially excluded groups including people experiencing homelessness is challenging and is central to elimination of TB in low incidence countries. These results show that an RRS is associated with improved TB treatment outcomes among these groups, which can inform national strategies to reduce and eliminate TB.

## Supplemental Material

sj-docx-1-rsh-10.1177_17579139221093544 – Supplemental material for Outcomes of a residential respite service for homeless people with tuberculosis in London, UK: a cross-sectional study Click here for additional data file. Supplemental material, sj-docx-1-rsh-10.1177_17579139221093544 for Outcomes of a residential respite service for homeless people with tuberculosis in London, UK: a cross-sectional study by L Crosby, D Lewer, Y Appleby, C Anderson, A Hayward and A Story in Perspectives in Public Health
