# Supplementary material for: Outcomes of a residential respite service for homeless people with tuberculosis in London, UK: a cross-sectional study
Source: Perspect Public Health. 2022 May 4;143(2):89–96. doi: 10.1177/17579139221093544 (PMC10068400; doi:10.1177/17579139221093544)
Supplement: sj-docx-1-rsh-10.1177_17579139221093544 – Supplemental material for Outcomes of a residential respite service for homeless people with tuberculosis in London, UK: a cross-sectional study [file sj-docx-1-rsh-10.1177_17579139221093544.docx]

**Supplementary information**

1. Logic model used to identify potential confounders of the association between treatment in the residential respite services and treatment outcomes
2. Detailed regression results
3. Simulation analysis
4. Sensitivity analysis

# Logic model used to identify potential confounders of the association between treatment in the residential respite services and treatment outcomes

Figure: logic model


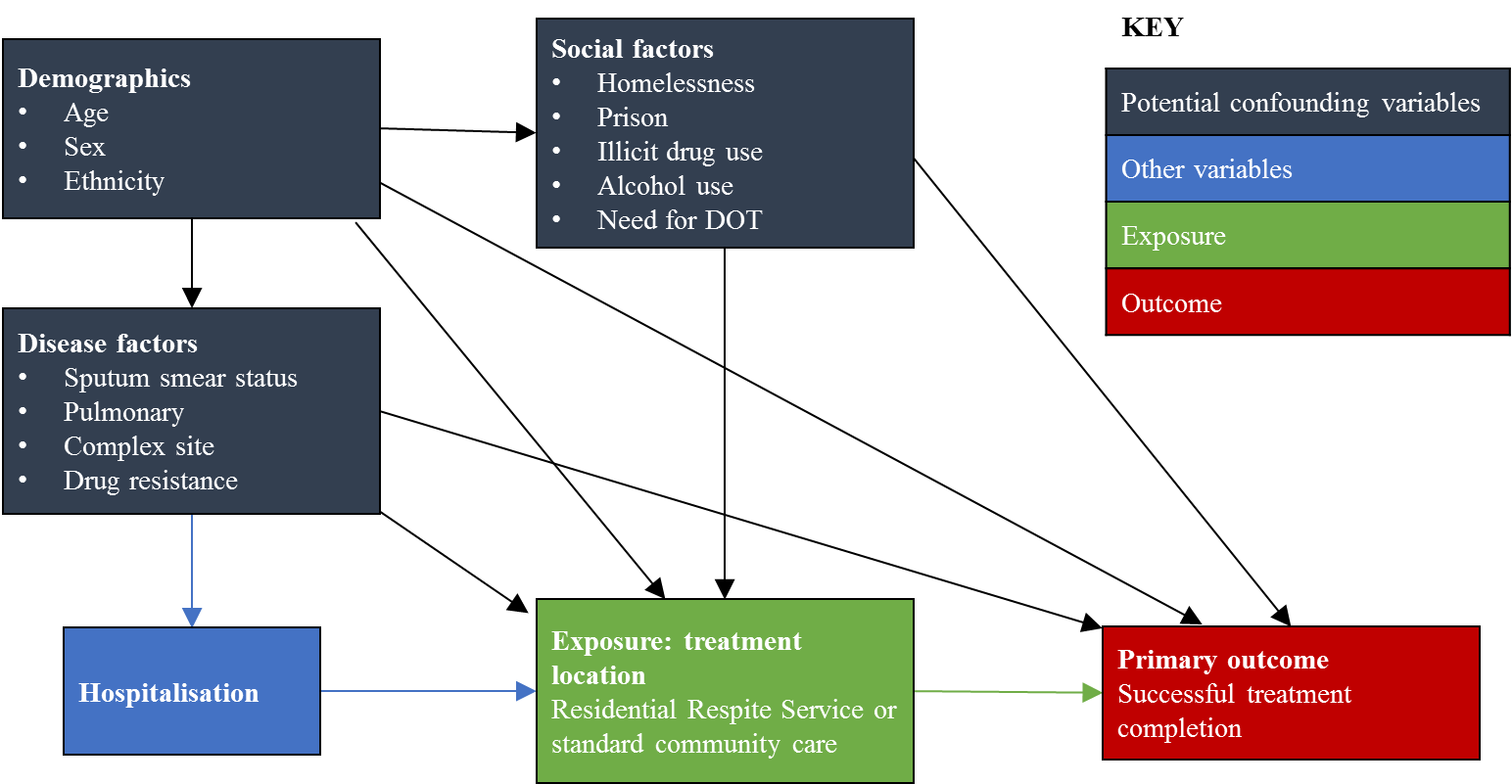


# Detailed regression results

Coefficients from our regression model are provided below. These effect sizes should be treated with caution because we have not considered the appropriate adjustment strategy for each variable (only treatment in the RRS).

Table: Likelihood of treatment completion among patients treated for tuberculosis in London, 2010-2019: detailed results of logistic regression

| **Variable** |  | **Crude odds ratio (95% CI)** | **Fully adjusted odds ratio (95% CI)** |
| --- | --- | --- | --- |
| Treated in RRS (ref: standard care) | | 1.03 (0.53-2.34) | 2.97 (1.44-6.96) |
| Age* |  | 0.70 (0.67-0.72) | 0.72 (0.69-0.76) |
| Female sex (ref: male) |  | 1.49 (1.36-1.63) | 1.29 (1.17-1.42) |
| Ethnicity | Asian (ref) | 1 | 1 |
|  | Black | 1.55 (1.38-1.75) | 1.81 (1.60-2.06) |
|  | White | 0.64 (0.57-0.72) | 1.01 (0.89-1.16) |
|  | Other | 1.18 (1.03-1.34) | 1.28 (1.11-1.47) |
|  | Unknown | 0.19 (0.14-0.25) | 0.54 (0.38-0.76) |
| Sputum smear positive (ref: negative) | | 0.66 (0.59-0.75) | 0.79 (0.68-0.92) |
| Pulmonary TB (ref: non-pulmonary) | | 0.62 (0.57-0.67) | 0.75 (0.67-0.82) |
| Complex site (ref: not complex site) | | 0.57 (0.48-0.68) | 0.64 (0.53-0.77) |
| Drug resistance | Full sensitive (ref) | 1 | 1 |
|  | Isoniazid mono-resistant | 0.67 (0.55-0.81) | 0.75 (0.61-0.93) |
|  | Rifampicin resistant or MDR | 0.46 (0.36-0.60) | 0.50 (0.38-0.66) |
| History of drug use | No (ref) | 1 | 1 |
|  | Yes | 0.55 (0.45-0.67) | 0.98 (0.78-1.25) |
|  | Missing | 0.13 (0.11-0.15) | 0.42 (0.32-0.55) |
| History of homelessness | No (ref) | 1 | 1 |
|  | Yes | 0.48 (0.40-0.57) | 0.88 (0.71-1.11) |
|  | Missing | 0.13 (0.11-0.15) | 0.83 (0.61-1.13) |
| History of prison | No (ref) | 1 | 1 |
|  | Yes | 0.44 (0.36-0.55) | 0.72 (0.56-0.92) |
|  | Missing | 0.11 (0.10-0.13) | 0.39 (0.29-0.52) |
| History of alcohol | No (ref) | 1 | 1 |
|  | Yes | 0.45 (0.38-0.54) | 0.98 (0.79-1.21) |
|  | Missing | 0.27 (0.24-0.31) | 0.81 (0.67-0.99) |
| Need for DOT recorded (ref: not recorded) | | 0.56 (0.50-0.61) | 0.62 (0.55-0.69) |

* Age is standardised such that the odds ratio represents the increase in the odds of treatment completion associated with an increase in one standard deviation of age

RRC = residential respite service (service for homeless patients with no recourse to public funds)

MDR = multi-drug resistant

DOT = directly observed treatment (often used for patients with high risk of not completing treatment)

# Simulation analysis

We then conducted a simulation to estimate how many treatment completions would be experienced among patients treated in the RRS if they were treated in standard community services (see Supplementary Information).

We fit a logistic regression model on the whole sample with treatment completion (primary outcome) as the dependent variable and the same independent variables as in our main regression model for primary outcome, but excluding the location of treatment. We then used this model to generate 1000 simulations of the primary outcome (i.e. treatment success/failure) for the 78 RRS patients with data on treatment completion; interpretable as scenarios in which these patients were treated in standard community care. We reported the 0.025, 0.5 and 0.975 quantiles of the number of treatment completions.

# Sensitivity analysis

In our primary analysis, where information on social risk factors (history of homelessness, drug use, prison or alcohol dependence) was missing, we coded the variable as ‘missing’.

We conducted a sensitivity analysis to assess the possible extent of bias resulting from this missing data. We created two scenarios: (1) imputing data for missing social risk factors for patients treated in the RRS as the presence of risk factors, and for patients treated in standard community care as the absence of risk factors, (2) the reverse scenario, imputing missing social risk factors for patients treated in the RRS as the absence of risk factors, and for patients treated in standard community care as the presence of risk factors. We reported the primary outcome in these scenarios.

In these sensitivity scenarios, the fully adjusted odds ratio was 2.87 (95% CI 1.40 to 7.03) in the first scenario and 3.00 (95% CI 1.48 to 6.97) in the second scenario, suggesting limited potential bias from missing data in social risk factors. However, we also observed strong associations between missing data and treatment failure, which may suggest a common process in which data is less likely to be recorded for patients who do not complete treatment. Coding these variables as ‘missing’, as in our main analysis, is therefore likely to be the most appropriate approach.
